# Mucormycosis in post-COVID patients: loose cannon waiting to fire at the anaesthesiologist

**DOI:** 10.1186/s42077-023-00331-9

**Published:** 2023-05-23

**Authors:** Avinash Prakash, Anita Yadav, Amrusha Raipure, Jyoti Baghel

**Affiliations:** 1 Department Of Anesthesiology, AIIMS Nagpur, Nagpur, Maharashtra India; 2 Department Of Obstetrics and Gynecology, AIIMS Nagpur, Nagpur, Maharashtra India

**Keywords:** Mucormycosis, COVOD-19, Difficult intubation, Anaesthetic challenge

## Abstract

**Background:**

Recently, several cases of mucormycosis in people with coronavirus disease 2019 (COVID-19) have been increasingly reported worldwide, in particular from India. As an increasing number of post-COVID patients with mucormycosis are presenting for surgical management, the onus is on the anaesthesiologists to adequately evaluate and optimise such patients.

**Case presentation:**

We present three cases to highlight the anaesthetic challenges in patients undergoing functional endoscopic sinus surgery (FESS) with debridement surgery for mucormycosis through a series of three cases. In our manuscript, two cases had comorbidities as a challenge uncontrolled diabetes and hypertension. The third case had difficult intubation due to a small mouth opening which was probably due to swelling and tenderness as a result of rhino orbital mucormycosis.

**Conclusions:**

Proper anticipation, optimization and timed promptness to deal with the anaesthetic challenges pertaining to the multisystemic involvement and sequelae of mucormycosis should be done.

## Background

Mucormycosis, also known as zygomycosis or phycomycosis is a fulminant and opportunistic fungal infection. The classical features of mucormycosis are angio-invasion, thrombosis, infarction and necrosis. Variations of mucormycosis include rhino-cerebral, pulmonary, gastrointestinal, cutaneous, and disseminated infections. Risk factors for such infections include diabetes mellitus, lymphoma, leukaemia, neutropenia, corticosteroid use, long-term immunosuppressive therapy and patients with thalassemia using desferrioxamine therapy (Petrikkos and Tsioutis 2018).

Recently, several cases of Mucormycosis in people with COVID-19 have been increasingly reported worldwide. The primary reason that appears to be facilitating Mucorales spore growth is an ideal environment of hypoxia, high glucose, acidic medium, increased ferritin levels and decreased phagocytic activity of white blood cells (Mishra et al. 2021). The mortality rates range from 16% in cutaneous mycosis up to 100% in gastrointestinal or disseminated infection with an overall mortality of 36–44% (Hingnikar et al. 2019).

## Case presentation

We here describe three cases of patients with COVID-19 infection, who during the course of the treatment, developed mucormycosis. Subsequently, the anaesthetic challenges in these patients undergoing functional endoscopic sinus surgery (FESS) with debridement for mucormycosis have been discussed.

### Case 1

**Fig. 1:**
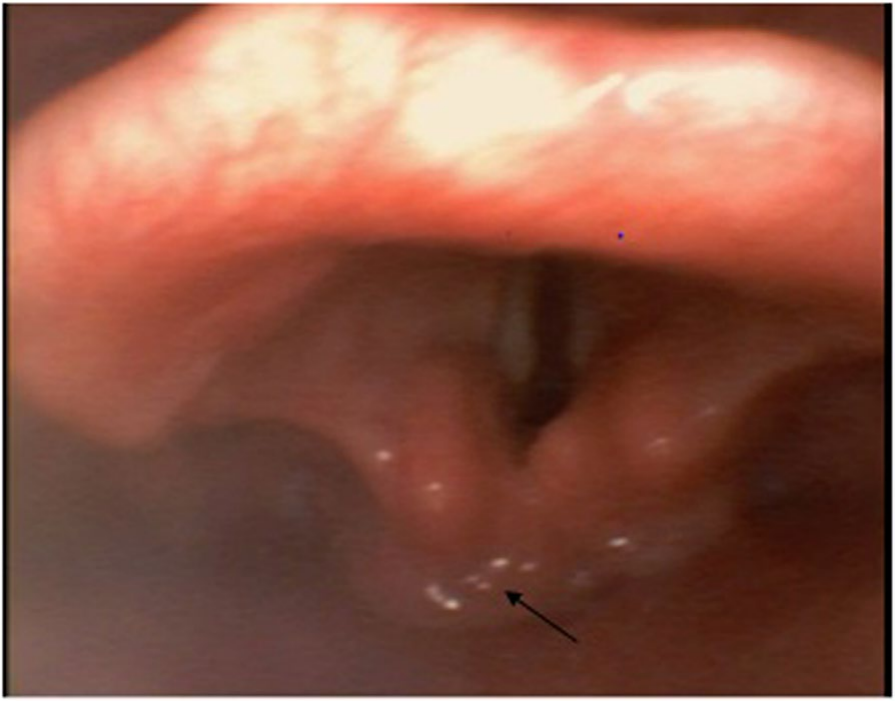
Fibreoptic bronchoscope shows floppy and oedematous epiglottis with mucor deposits (marked with arrow)

A 64-year-old female, known diabetic and hypertensive, presented with complaints of fever, right-sided progressive headache, pain and swelling in the right eye for 10 days. She tested COVID-19 positive 2 months back. Pre-anaesthetic (PAC) evaluation revealed a pulse rate (PR) of 120 per minute and blood pressure (BP) of 150/90 mm of mercury (mm Hg). The thyromental distance was 6.5 cm (cm). Magnetic resonance imaging (MRI) of the brain, orbits, and paranasal sinuses (PNS), showed soft tissue swelling in the right pre-septal, malar, premaxillary and retrobulbar regions. Inj dexmedetomidine 1 mcg/kg IV infusion started and was given for 10 min then switched over to maintenance. The dose was 0.2 to 0.6 mcg/kg/h IV infusion; the infusion rate was adjusted to achieve the desired clinical effect i.e. target blood pressure below 110/70 mmHg. In this case, the problem encountered was bag-mask ventilation after induction of anaesthesia, which was taken care of by using an oral airway and with the help of one more qualified anaesthesiologist to assist in bag mask ventilation. Direct laryngoscopy was performed using a Macintosh 4 blade on which the epiglottis appeared swollen and floppy (Cormack Lehane (CL) Grade II B). With the help of video-laryngoscope tracheal and BURP, intubation was done under vision with 7.5 mm (mm) cuffed reinforced endotracheal tube. The epiglottis was oedematous, fungal deposits were seen in the vallecula as shown in Fig. 1. Intraoperative procedure went uneventful, and hypertension was controlled within the normal range with the help of a titrating dose of dexmedetomidine and inhalational agent. Dexmedetomidine was stopped about 20 min prior to the end of surgery and extubation. Bispectral index (BIS) monitoring and neuromuscular monitoring were done to maintain adequate depth of anaesthesia. To assure complete and timely recovery from anaesthesia and to avoid hypertension smooth reversal was done, extubated gently following BIS value preferably when the patient was a bit drowsy and started generating a tidal volume of 5–7 ml/kg and started obeying verbal commands.

### Case 2

Fifty-nine-year-old male with a history of COVID-19 infection presented with complaints of epistaxis, decreased vision and dental pain for 3 days. He was on non-invasive ventilation during the course of his COVID-19 illness. He had a history of uncontrolled diabetes (glycated haemoglobin [HbA1c] was 10.2%) and received steroids for 10 days. Furthermore, his PAC evaluation showed stable vitals with an oxygen saturation level (SPO2) of 88–90% on room air. Mouth opening was > 3 cm and thyromental distance > 6 cm. Breath-holding time 18–20 s. MRI PNS showed mucosal thickening in bilateral maxillary and anterior and posterior ethmoidal walls. Uncontrolled diabetes was the challenge in this case. Diabetic ketoacidosis was ruled out. Neutralising dose of 12 units of insulin in 500 ml of 5% dextrose was started at the rate of 14–16 drops per min, and for strict blood sugar control, intraoperatively hourly random blood sugar (RBS) level was monitored and insulin infusion was titrated accordingly. A maintenance infusion of potassium chloride (KCL) was started and continued intraoperatively at the rate of 2.5 ml per hour. Postoperatively blood sugar and serum electrolyte levels were monitored and corrections were supplemented as per reports.

### Case 3

A 50-year-old man with a history of COVID-19 infection presented with left eye swelling and pain for five days. Preanesthetic evaluation showed stable vitals. Mouth opening was about 1.5 cm, Mallampati classification (MPC III) and thyromental distance ≥ 6 cm. Breath-holding time was 12–16 s. MRI PNS imaging is suggestive of mucosal thickening and sinusitis. Here, difficult intubation was anticipated due to a small mouth opening (1.5 cm). Small mouth opening was probably due to swelling and tenderness around the left eye as well as the left infraorbital area of the face. Fibreoptic bronchoscope and video laryngoscope were kept ready for intubation. The patient was counselled for awake intubation. Injection Glycopyrrolate 0.2 mg was intravenously given once patient was to be shifted to OT. After preparing the airway, an injection of dexmedetomidine 0.5 mcg per kg was started intravenously through an infusion pump for sedation and anxiolysis. Once the patient became calm and a bit sleepy, then intubation was done gently with the help of a fibreoptic bronchoscope.

## Discussion

Successful treatment of mucormycosis is dependent on four key principles: early diagnosis, treatment of underlying predisposing factors, surgical debridement of necrotic tissue, and administration of antifungal therapy (Karaaslan 2019). Routine monitoring including heart rate (HR), non-invasive blood pressure (NIBP), electrocardiogram (ECG), end-tidal CO_2_ concentration and peripheral oxygen saturation (SpO2) should be done for each patient. For patients with deranged renal and liver functions, bispectral index (BIS) monitoring and neuromuscular monitoring is vital to maintain adequate depth of anaesthesia and assure complete and timely recovery from anaesthesia. Post-COVID, the chances of postoperative respiratory complications are high for up to 4–12 weeks of illness. The lesser the duration between the onset of COVID symptoms and the requirement of anaesthesia for surgery, the higher will be the perioperative morbidity and mortality (Bui et al. 2021). Assessing the effort tolerance of the patient, a 6-min walk test, breath-holding time, and ambulatory oxygen saturation are some simple ways to evaluate the cardiopulmonary status. A preoperative 2D Echocardiography may be considered depending on the patient’s clinical evaluation.

The associated co-morbidities need to be evaluated and optimised within the available time frame before taking up for surgery. One of the most common co-morbidities in these patients is diabetes mellitus which is often uncontrolled and requires strict vigilance. The sugars need to be monitored regularly and frequently require treatment with Insulin infusion to assure an immediate peri-operative period. Continuous measurement of arterial blood gas values and intermittent biochemical analyses are essential for the management of the metabolic condition, fluid-electrolyte balance, and coagulopathy peri-operatively (Karaaslan 2019).

**Fig. 2:**
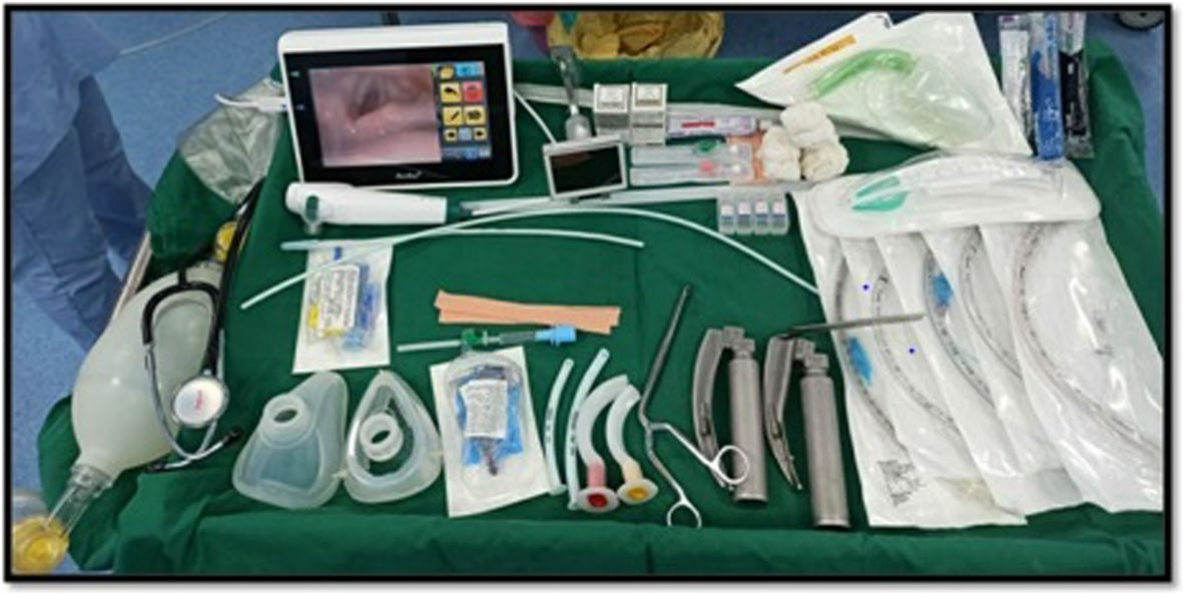
Difficult intubation cart

Patients with Rhino-orbital-cerebral (ROC) mucormycosis involvement may experience difficult mask ventilation and endotracheal intubation as a result of epiglottitis and supraglottic oedema associated with fungal debris. Anaesthesiologists should verify that the operating theatre has adequate facemasks, intubating stylets, tube exchange or gum elastic bougies, laryngeal mask airways, video laryngoscopes, rigid laryngoscope blades of varying designs or sizes, fibreoptic bronchoscope, wide bore suction and emergency tracheotomy for unexpectedly difficult airway management. A difficult intubation cart should be always kept ready as shown in Fig. 2.

Attending anaesthesiologists should have knowledge of intravenous amphotericin B (AmB) induced renal and cardiac changes and their interactions. AmB-induced hypokalaemia may enhance the effect of skeletal muscle relaxants. When administered concurrently, serum potassium levels should be monitored. Arrhythmia, atrial fibrillation, bradycardia, cardiac arrest, cardiomegaly, haemorrhage, postural hypotension, and vasodilatation have been reported (Kulkarni et al. 2015). Concurrent use of insulin and digitalis glycosides may potentiate hypokalaemia, which could predispose the patient to cardiac dysfunction and digitalis toxicity. Serum electrolytes and cardiac function should be monitored in such cases.

Fluid replacement, blood and blood products transfusion and intraoperative ionotropic support may be required in some patients, which may necessitate central venous cannulation. Interventional procedures should not be performed at a location close to the infected site since these patients are at increased risk of rapid development of immunosuppression, neutropenia, and thrombocytopenia.

## Conclusions

Proper anticipation, optimization through medication, supplements, another qualified anaesthesiologist as a backup and prompt readiness to deal with the anaesthetic challenges can ameliorate the outcome of patients’ peri- and post-surgery.

## Data Availability

Available with the corresponding author.
